# Elevation in Body Temperature to Fever Range Enhances and Prolongs Subsequent Responsiveness of Macrophages to Endotoxin Challenge

**DOI:** 10.1371/journal.pone.0030077

**Published:** 2012-01-10

**Authors:** Chen-Ting Lee, Lingwen Zhong, Thomas A. Mace, Elizabeth A. Repasky

**Affiliations:** Department of Immunology, Roswell Park Cancer Institute, Buffalo, New York, United States of America; University of Miami School of Medicine, United States of America

## Abstract

Macrophages are often considered the sentries in innate immunity, sounding early immunological alarms, a function which speeds the response to infection. Compared to the large volume of studies on regulation of macrophage function by pathogens or cytokines, relatively little attention has been devoted to the role of physical parameters such as temperature. Given that temperature is elevated during fever, a long-recognized cardinal feature of inflammation, it is possible that macrophage function is responsive to thermal signals. To explore this idea, we used LPS to model an aseptic endotoxin-induced inflammatory response in BALB/c mice and found that raising mouse body temperature by mild external heat treatment significantly enhances subsequent LPS-induced release of TNF-α into the peritoneal fluid. It also reprograms macrophages, resulting in sustained subsequent responsiveness to LPS, i.e., this treatment reduces “endotoxin tolerance” *in vitro* and *in vivo*. At the molecular level, elevating body temperature of mice results in a increase in LPS-induced downstream signaling including enhanced phosphorylation of IKK and IκB, NF-κB nuclear translocation and binding to the TNF-α promoter in macrophages upon secondary stimulation. Mild heat treatment also induces expression of HSP70 and use of HSP70 inhibitors (KNK437 or Pifithrin-µ) largely abrogates the ability of the thermal treatment to enhance TNF-α, suggesting that the induction of HSP70 is important for mediation of thermal effects on macrophage function. Collectively, these results support the idea that there has been integration between the evolution of body temperature regulation and macrophage function that could help to explain the known survival benefits of fever in organisms following infection.

## Introduction

A primary function of macrophages is to remove cellular debris generated during normal tissue function while following tissue injury or infection, macrophages can respond rapidly to various “alarm” signals generated from inflamed sites and become activated to release pro-inflammatory mediators [1], [2], [3]. Current information suggests that macrophages are optimally activated by a combination of two macromolecular signals in their environment: antigen recognition by pattern recognition receptors and IFN-γ [4], [5]. Production of pro-inflammatory cytokines by resident macrophages in turn promotes the recruitment of neutrophils and inflammatory monocytes/macrophages to the inflamed region [6]. Integrated actions among these immune phagocytes help to eliminate pathogens, repair damaged tissues, initiate an adaptive immune response, and most importantly, restore tissue homeostasis [6], [7], [8].

While there has been much attention devoted to the pathogens, receptors and cytokines which help to activate and regulate macrophages during inflammation, comparatively little work has examined the role of the physical aspects of the microenvironment, such as temperature, in the regulation of macrophage function. Fever, which is widely recognized as an elevation in body temperature, is a highly-conserved “cardinal sign” of infection and inflammation in both endotherms and ectotherms. While endotherms have the ability to dynamically regulate their metabolism to increase their body temperatures, ectotherms must rely largely on external warmth to raise their body temperature [9], [10], [11], [12], [13]. While the biological significance of fever is a topic of long-lasting debate, it is important to note that many studies have revealed a significantly positive relationship between elevated temperatures and improved survival rate following infection [9], [14], [15], [16]. It is clear from these earlier studies that the improved survival seen following fever is not simply due to thermal suppression of bacterial growth [16].

Previous data published by our laboratory [17] have demonstrated that mild systemic heat treatment, raising core temperature to 39.5°C, significantly enhances the concentration of TNF-α and IL-6 in the serum of BALB/c mice challenged with LPS. Jiang et al. [18], [19] identified macrophages as the predominant source of increased circulating TNF-α in the serum of warmed animals. These studies provide important clues suggesting that febrile temperatures may specifically modulate macrophage function. However, much more work is needed to clarify the mechanisms in which thermal signals regulate macrophage function in response to LPS challenge.

In this current study, we investigated possible cellular and molecular mechanisms by which heat treatment affects macrophage cytokine production using a mouse model of LPS-induced aseptic inflammation. Moreover, for the first time, we have explored the effect of fever-range temperatures on macrophage cytokine production after LPS rechallenge and on induction of “endotoxin tolerance”.

## Results

### Heat treatment increases LPS-induced TNF-α production *in situ* by peritoneal macrophages

**Figure 1:**
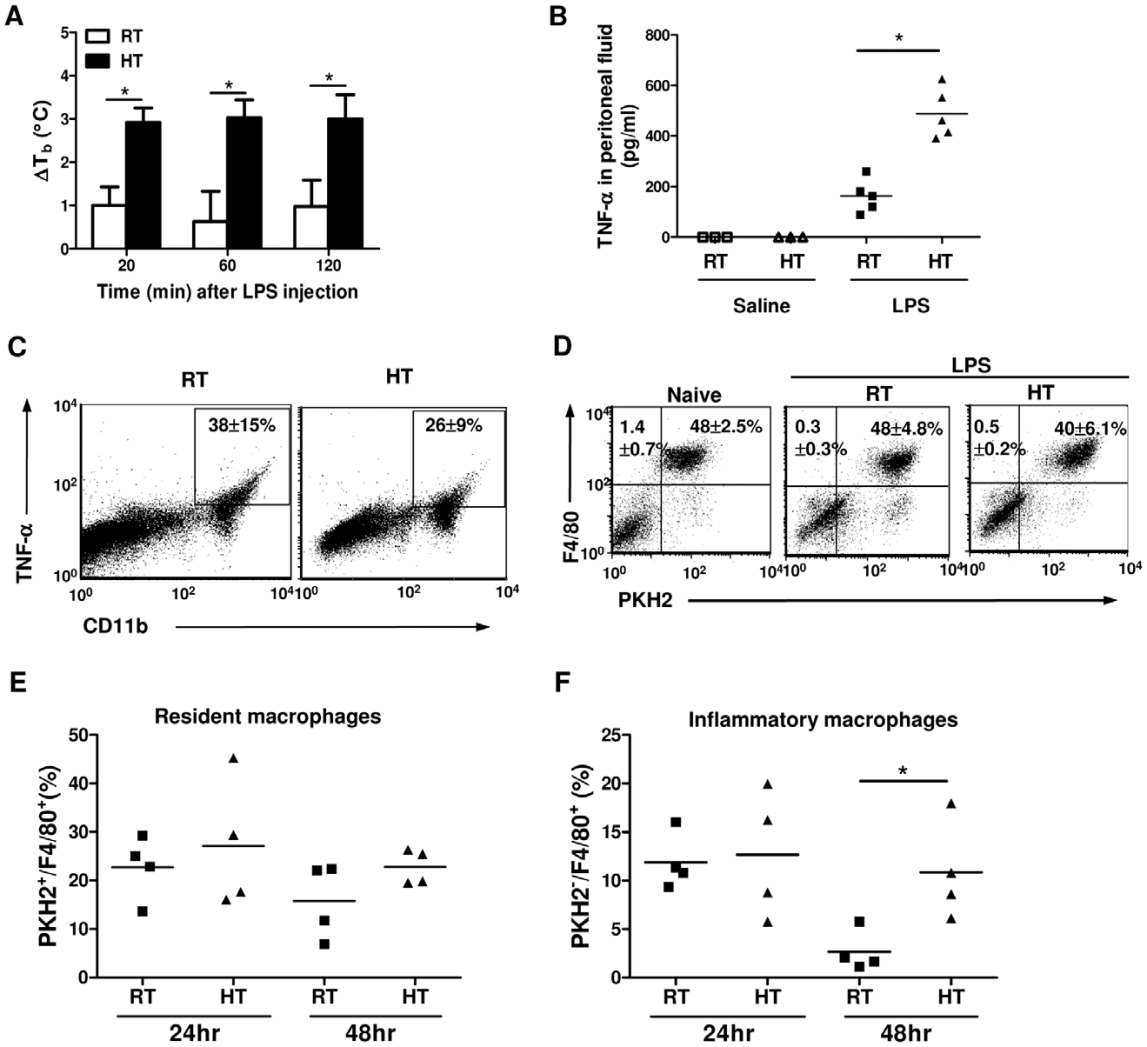
Heat treatment increases LPS-induced TNF-α production. *A*, BALB/c mice were injected intraperitoneally with 10 µg of LPS and then received heat treatment (HT, 39.5°C) immediately or were kept at room temperature (RT) for 2 hours. The core body temperature of these mice was measured prior to and 20, 60 and 120 minutes after LPS injection and changes in the body temperature (ΔT_b_) were shown. Baseline temperatures of these mice were 36.2 ±0.6°C. Data are mean± SD (n = 4 mice per treatment groups). *B*, Peritoneal fluids were collected 2 hours post injection from LPS-challenged or saline-injected mice and TNF-α concentration was determined by ELISA. Each symbol represents an individual mouse (n = 3–5 mice per treatment groups). *C*, Peritoneal cells were collected 2 hours post injection from LPS-challenged mice and stained with CD11b and TNF-α mAb for intracellular TNF-α staining. The numbers represent the percentage of TNF-α^+^ cells within CD11b^+^ cells. Data are mean ± SD. *D* to *F*, BALB/c mice were injected intraperitoneally with the fluorescent dye PKH2 to label resident peritoneal macrophages *in vivo* two days before LPS challenge. Mice were then injected with 10 µg LPS and received HT immediately or were kept at RT for 2 hours. Peritoneal cells were collected from these mice 2 hours (*D*), 1 and 2 days (*E*–*F*) after, stained with F4/80 mAb and analyzed by flow cytometry to determine the percentage of PKH2^+^F4/80^+^ resident and PKH2^−^F4/80^+^ inflammatory macrophages. Each symbol represents an individual mouse. Data are representative of two independent experiments. *p<0.05; paired Student *t* test.

We and others have previously reported that mild heating of mice significantly enhances the concentration of LPS-induced pro-inflammatory cytokines in the serum [17], [18], [19]. To identify the cellular source of cytokine production *in vivo*, we injected LPS intraperitoneally into BALB/c mice which then received heat treatment (HT) to help increase their body temperature or into mice which were maintained under standard room temperature conditions (RT, ∼22–24°C) for 2 hours. Even though LPS can be a pyrogen itself, at the dose used here and with no provision of additional ambient warmth, we only observed a modest elevation of body temperature of 1°C or less after LPS injection in RT mice (Fig. 1*A*). On the other hand, as demonstrated by Jiang et al. [18], providing additional HT can raise the core temperature to 39–39.5°C (Fig. 1*A*). We then measured the level of TNF-α in the peritoneal fluid by ELISA. Fig. 1*B* revealed that in LPS-challenged mice, HT significantly increased the concentration of TNF-α *in situ* whereas TNF-α was undetectable in the peritoneal fluid of saline-injected naive mice that received either HT alone or were kept at RT. Macrophages are known to be the predominant source of TNF-α production in the peritoneal cavity. To determine their role in our model, we isolated peritoneal cells from mice after 2 hours LPS stimulation and analyzed TNF-α production by intracellular staining. Our data showed that all the TNF-α producing cells were within the CD11b^+^ macrophage population (Fig. 1*C*).

Since macrophages play an important role in pathogen clearance and resolution of inflammation, we next investigated whether HT affected macrophage recruitment after LPS stimulation. We administered the fluorescent dye PKH2, which was taken up specifically by phagocytes *in situ*, into mice to label resident peritoneal macrophages two days before the LPS challenge. This dye uptake protocol was published and demonstrated to distinguish resident from recruited inflammatory macrophages [20], [21], [22], [23]. In naïve mice, resident macrophages, which were shown as PKH2^+^ F4/80^+^ cells, represented approximately 50% of the total peritoneal cells (Fig. 1*D left panel*). In mice challenged with LPS for two hours, this percentage did not change as compared to cells from naive mice. Furthermore, LPS-challenged mice receiving HT exhibited a similar percentage and total cell number of resident macrophages compared to those from untreated mice (Fig. 1*D* *middle and right panels* and data not shown), demonstrating that HT has no effects on macrophage recruitment two hours after LPS exposure. To study the long term effect of HT, we isolated peritoneal cells from LPS-challenged mice one and two days after LPS injection. We observed a trend toward a decrease in the percentage of resident macrophages (PKH2^+^ F4/80^+^) with time in both RT and HT groups. However, this decrease did not reach statistical significance (Fig. 1*E*). On the other hand, we observed more inflammatory macrophages (PKH2^−^ F4/80^+^) in the heat-treated mice two days after LPS injection as compared to mice maintained at RT (Fig. 1*F*). However, there was only a small difference in the cell number of inflammatory macrophages due to individual variances existing in these mice and small sample size (Fig. S1).

### Macrophages from heat-treated mice produce more pro-inflammatory cytokines after *in vitro* re-stimulation

**Figure 2:**
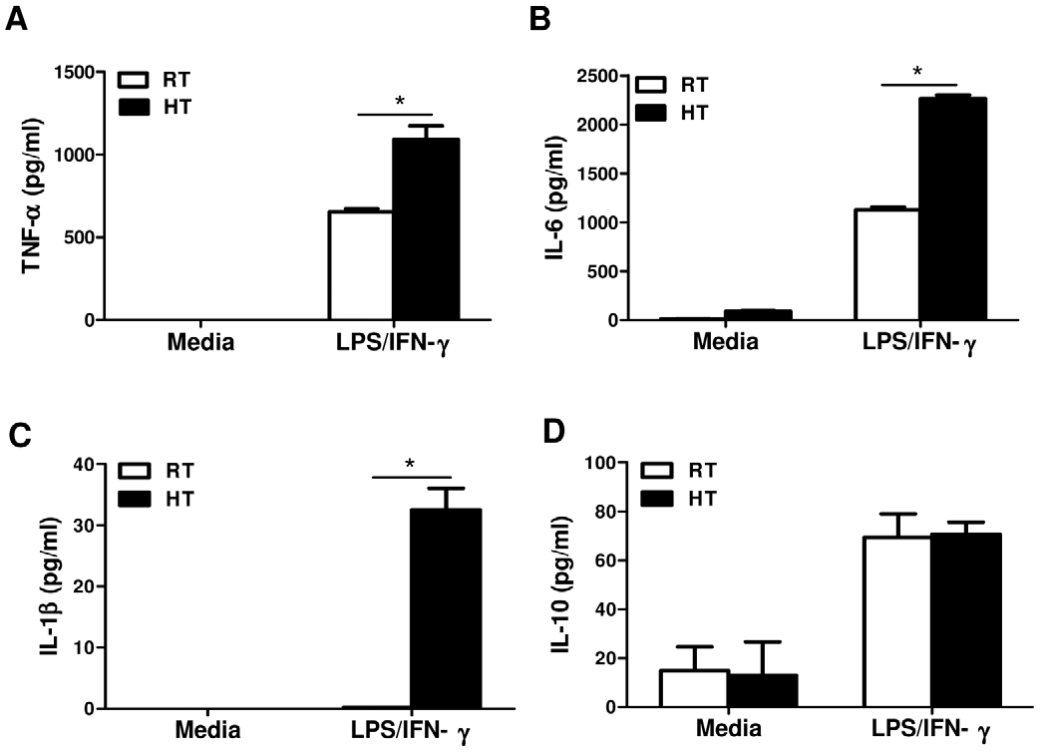
Macrophages from heat-treated mice produce more pro-inflammatory cytokines after *in vitro* re-stimulation. *A* to *D*, BALB/c mice were injected with 10 µg of LPS and then received HT or were kept at RT for 2 hours. Peritoneal macrophages were isolated from these mice 2 hours post injection, recovered overnight and re-stimulated (2×10^5^/well) with LPS (100 ng/mL) and IFN-γ (25 U) *in vitro* at 37°C for 6 hours to determine TNF-α (*A*), IL-6 (*B*), and IL-10 (*D*) or 24 hours for IL-1β (*C*) production by ELISA. Cells from each treatment condition were pooled from 2 mice and measured in triplicate. Data are mean ± SD. Data are representative of three independent experiments. *p<0.05; paired Student *t* test.

To further determine how the thermal microenvironment affects macrophage function, we isolated peritoneal macrophages two hours post injection from LPS-challenged mice which had or had not received HT, waited 24 hours to allow for a recovery period from the isolation process and then analyzed their cytokine production *in vitro*. Our data showed that these macrophages did not secrete TNF-α without *in vitro* re-stimulation (Fig. 2*A*). With *in vitro* LPS/IFN-γ re-stimulation, macrophages isolated from heat-treated mice produced higher levels of TNF-α as compared to those cells isolated from mice kept at RT (Fig. 2*A*). HT also enhanced macrophage IL-6 and IL-1β production (Fig. 2*B and 2C*). On the other hand, there was no difference in anti-inflammatory cytokine IL-10 production between macrophages isolated from heat-treated and RT mice (Fig. 2*D*). We also measured TNF-α production by peritoneal macrophages from naïve mice with or without HT. LPS/IFNγ stimulation induced TNF-α production in these cells, but in contrast to macrophages from LPS-challenged mice, macrophages from naïve heated mice showed no enhancement of TNF-α production over the cells from unheated mice (Fig. S2). This indicates that heat treatment alone, without LPS stimulation, does not affect subsequent cytokine production by naïve macrophages.

### Macrophages from heat-treated mice exhibit more TNF-α producing cells as well as a higher level of TNF-α production on a per cell basis after *in vitro* re-stimulation

**Figure 3:**
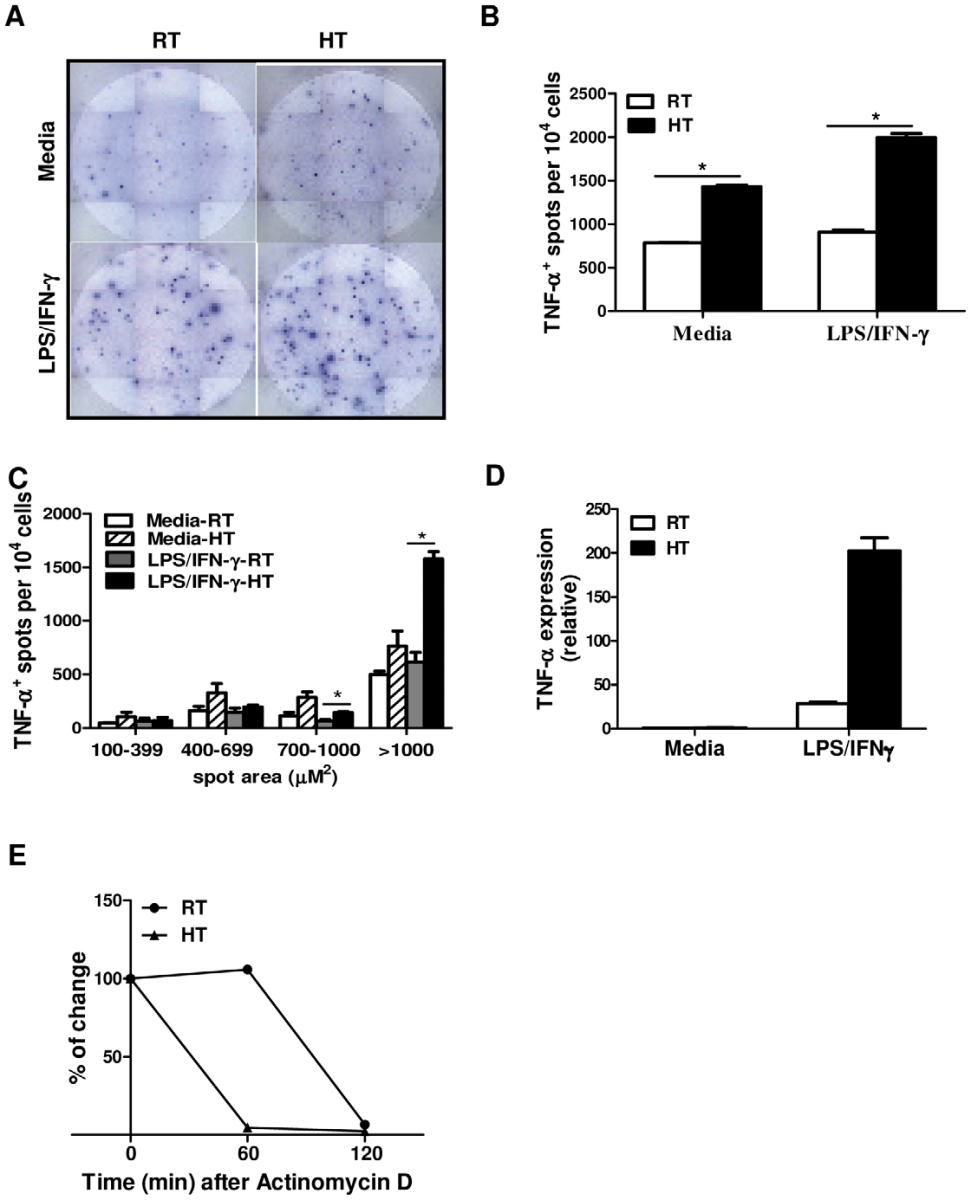
Effects of heat treatment on the number of TNF-α producing cells and LPS-induced TNF-α gene expression. *A* to *C*, Peritoneal macrophages were isolated 2 hours post injection from LPS-challenged mice with or without HT, recovered overnight and re-stimulated with LPS and IFN-γ at 37°C for 24 hour. TNF-α producing cell was measured by ELISpot assay. The absolute number of TNF-α-producing macrophages was calculated per 10^4^ macrophages (*B*) or calculated based on different sizes of spot area (*C*) by the Carl Zeiss Vision EliSPOT software. Data are mean ± SD. Cells from each treatment condition were pooled from 4 mice. Data are representative of two independent experiments. *p<0.05; paired Student *t* test. *D*, Peritoneal macrophages were isolated from LPS-challenged mice as mentioned before, allowed to recover overnight and then re-stimulated (1×10^6^/well) with LPS and IFN-γ at 37°C for 4 hours to measure TNF-α mRNA by quantitative real-time PCR. The results are presented relative to GAPDH and baseline expression in unstimulated cells from RT-mice. *E*, RNA decay analysis was performed by using peritoneal macrophages (1×10^6^/well) from LPS-challenged mice that were re-stimulated with LPS and IFN-γ at 37°C for 2 hours. Actinomycin D (5 µg/ml) was then added to block further transcription. Total RNA was extracted at different time points after actinomycin D blocking and then analyzed for TNF-α mRNA expression by quantitative real-time PCR. Data represents the percent change of TNF-α mRNA compared to the cells before actinomycin D treatment. Data are measured in duplicate by Q-PCR.

To determine if HT affects the numbers of TNF-α producing macrophages or TNF-α production on a per cell basis after *in vitro* re-stimulation, a TNF-α ELISpot was performed (Fig. 3*A*–*C*). Both before and after *in vitro* LPS/IFN-γ re-stimulation, the numbers of TNF-α secreting macrophages were increased in LPS-challenged, heated mice as compared to cells from RT mice (Fig. 3*A*–*B*). We further analyzed the amount of TNF-α production per cell based on the spot area. Our results showed that HT also significantly increased the population of cells that produced high amounts of TNF-α. There were more TNF-α^+^ cells with spot area more than 1000 µM^2^ in heat-treated, LPS/IFN-γ re-stimulated macrophages than that from other treatment groups (Fig. 3*C*). Hence, after *in vitro* LPS re-exposure, this thermally-enhanced TNF-α production is due to an increase in the number of TNF-α producing macrophages, especially the population of cells that secrete high amounts of TNF-α.

### Macrophages from heat-treated mice have an increased TNF-α mRNA expression after *in vitro* re-stimulation

To assess the molecular mechanisms of how the thermal signal enhances macrophage cytokine production after LPS re-exposure, we first determined whether prior HT altered subsequent macrophage TNF-α gene transcription by measuring TNF-α mRNA levels by quantitative real-time PCR. Our results showed that macrophages from heat-treated mice had an increased TNF-α mRNA induction approximately 200 fold after *in vitro* LPS/IFN-γ re-stimulation as compared to cells from RT mice (Fig. 3*D*). To determine if HT changes TNF-α mRNA stability, we re-stimulated these macrophages with LPS/IFN-γ for 2 hours and then added actinomycin D to block transcription. We found that in macrophages isolated from control mice, TNF-α mRNA started to decay 1 hour after the addition of actinomycin D (Fig. 3*E*). On the other hand, TNF-α mRNA decayed more rapidly in macrophages isolated from heat-treated mice. These data suggest that the thermally-enhanced TNF-α mRNA expression is not due to an increase in TNF-α mRNA stability but likely reflect an increase in TNF-α gene transcription.

### Heat treatment enhances macrophage NF-κB activation in response to LPS re-stimulation

**Figure 4:**
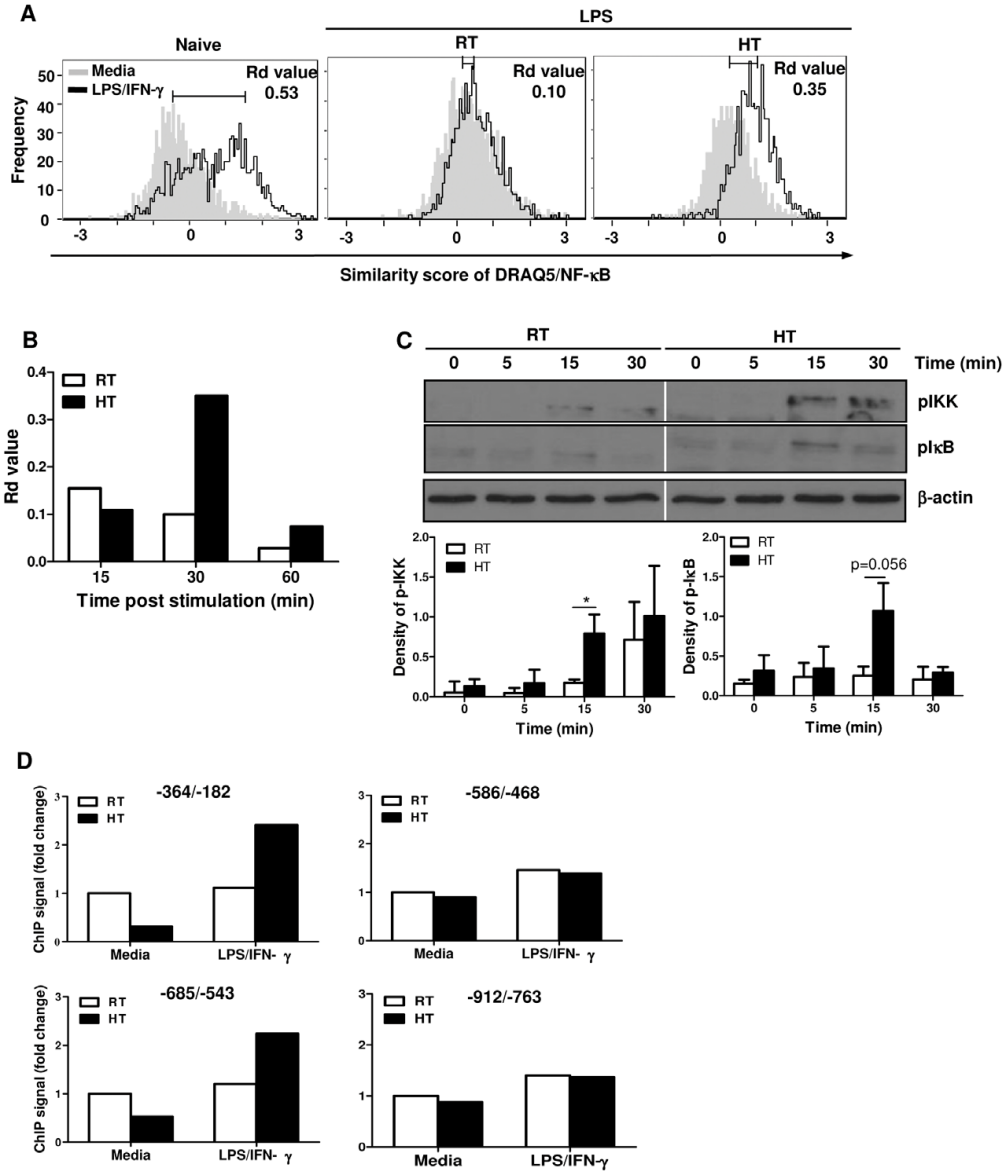
Peritoneal macrophages from LPS-challenged, heat-treated mice show more NF-κB activation after *in vitro* LPS/IFN-γ re-stimulation. *A* to *B*, Peritoneal macrophages were isolated from naïve or LPS-challenged, RT or heat-treated mice, recovered overnight and stimulated (1×10^6^/well) *in vitro* with LPS/IFN-γ at 37°C for 30 min (*A*) or indicated time points (*B*), stained with antibodies against CD11b, NF-κB p65 and DRAQ5 DNA dye and then analyzed by ImageStream flow cytometry. CD11b^+^ cells were gated to show the similarity score between DRAQ5 nuclear staining and NF-κB staining. Rd value = (similarity score of treated - untreated)/(standard deviation of the similarity score from treated + untreated). *C*, Macrophages (1×10^6^/well) from LPS-challenged, RT or heat-treated mice were re-stimulated *in vitro* with LPS/IFN-γ at 37°C for 0, 5, 15 and 30 min to detect the phosphorylation of IKK and IκB by Western blotting. The graph shows the quantification of the band intensity of pIKK and pIκB normalized to β-actin. Data are mean ± SD from three independent experiments. *D*, Macrophages (1×10^6^/well) from LPS-challenged, RT or heat-treated mice were stimulated *in vitro* with LPS/IFN-γ at 37°C for 30 min. Cross-linked chromatin was immunoprecipitated with anti-NF-κB p65 antibody and analyzed for NF-κB p65 binding to the TNF-α promoter region by quantitative real-time PCR with primers spanning the regions −364/−182, −586/−468, −685/−543 and −912/−763. The graph shows the fold change which is normalized to the input and control IgG and then compared with unstimulated or RT-unstimulated cells. Cells from each treatment condition were pooled from 4 mice. Data are representative of two independent experiments.

TLR4 is the major pattern recognition receptor for LPS and triggers signals which activate macrophages [24]. To investigate the molecular changes associated with thermally-enhanced TNF-α production, we examined whether HT directly modulated LPS-induced TLR4 signaling pathway in macrophages. While our data showed an increase in TLR4 expression on macrophages from heat-treated mice as compared to cells from RT and naïve mice (Fig. S3), this increase did not reach statistical significance. Since there was only a modest increase in TLR4, we decided to focus on the effect of HT on LPS-induced downstream signaling and NF-κB activation. Upon LPS stimulation, sequestered latent cytoplasmic NF-κB is released, activated and translocated into the nucleus where it binds to the promoter region of the target genes and activates their transcription [25]. To investigate NF-κB activation in macrophages, we used ImageStream flow cytometry to quantify the degree of NF-κB nuclear translocation by calculating the similarity of NF-κB and nuclear dye DRAQ5 images as shown in Fig. 4A. The relative shift in the distribution between cells before and after stimulation was calculated using the Fisher's Discriminant ratio (*R*_d_ value) [26]. Higher *R*_d_ value indicated more NF-κB was translocated into the nucleus (Fig. 4*A*). In naïve macrophages, LPS/IFN-γ stimulation increased NF-κB nuclear translocation as compared to the cells without stimulation (Fig. 4*A left panel*). To determine whether HT affects NF-κB activation upon secondary stimulation, we re-stimulated macrophages from LPS-challenged, heated and RT-mice with LPS/IFNγ at 37°C for 15, 30 and 60 minutes. We found that macrophages from heat-treated mice had more NF-κB nuclear translocation 30 and 60 minutes after *in vitro* LPS/IFN-γ re-stimulation as compared to cells from RT-mice (Fig. 4*A* *middle, right panels and 4B*). Higher basal level of NF-κB nuclear translocation was observed in the macrophages isolated from the LPS-challenged mice as compared to those from naïve mice (Fig 4*A* *middle* and *right* vs. *left panels*). It is likely that macrophages from LPS-challenged mice had already been activated *in vivo*.

IKK/IκB pathway plays an important role in mediating the activation of NF-κB in macrophages [27]. IKK (IκB kinase) can be phosphorylated and activated by signals triggered by LPS, leading to phosphorylation and degradation of IκB, which is an inhibitory molecule binding to NF-κB at resting state. To evaluate whether NF-κB activation is affected by HT through the IKK/IκB pathway, macrophages from LPS-challenged, RT or heat-treated mice were re-stimulated *in vitro* by LPS/IFNγ at 37°C for 5, 15, and 30 minutes. Subsequent phosphorylation of IKK and IκB was detected by immunoblotting. The results showed that phosphorylation of both IKK and IκB after re-stimulation was enhanced by the prior HT (Fig. 4*C*), indicating that the signals transmitted through IKK/IκB pathway are increased by heat treatment.

Next, we determined whether the binding of NF-κB to TNF-α promoter was affected by HT using ChIP assay. The murine TNF-α promoter contains 4 κB binding sites located at 210, 510, 655 and 850 nucleotide upstream of the transcription start site [28]. In naïve macrophages, LPS/IFN-γ stimulation resulted in an increased binding of NF-κB to all the κB sites in the TNF-α promoter region (Fig. S4). After secondary stimulation, there was no increase of NF-κB binding to the TNF-α promoter in macrophages from LPS-challenged RT mice (Fig. 4*D*). However, HT resulted in an increased binding of NF-κB to the two κB sites contained in the two sequences −364/−182 and −685/−543 in the TNF-α promoter region, whereas there was no change in NF-κB binding on the other two sites, −586/−468 and −912/−763 (Fig. 4*D*). Overall, these results demonstrate that heat treatment enhances LPS-induced NF-κB activation in macrophages upon secondary stimulation which may lead to an increase of TNF-α gene transcription.

### HSP70 plays a role in mediating thermally-enhanced TNF-α production in macrophages

**Figure 5:**
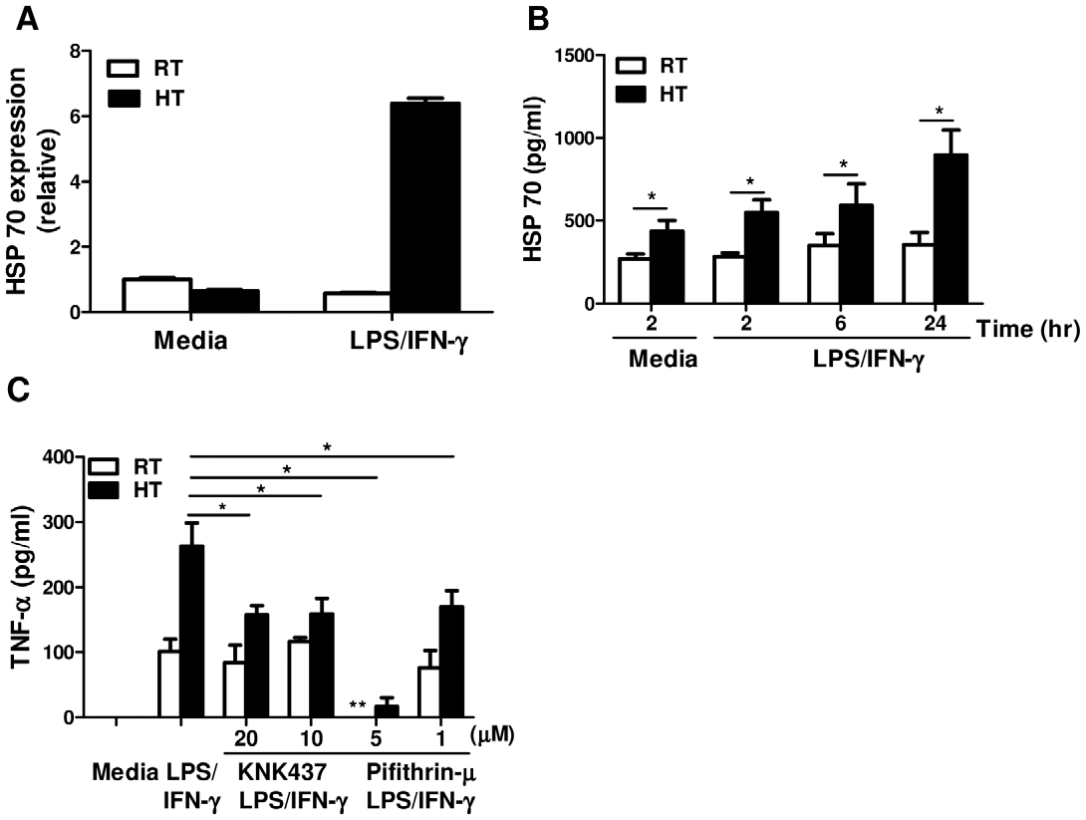
HSP70 plays a role in mediating thermally-enhanced TNF-α production in macrophages. *A*, Peritoneal macrophages were isolated from LPS-challenged mice after 2 hour heat treatment. Cells were recovered overnight and re-stimulated (1×10^6^/well) *in vitro* with LPS and IFN-γ at 37°C for 4 hours, then HSP70 mRNA level was measured by quantitative real-time PCR. The results are presented relative to GAPDH and baseline expression in unstimulated cells from RT-mice. *B*, Macrophages (2×10^5^/well) from LPS-challenged mice were re-stimulated with LPS and IFN-γ at 37°C for 2, 6 or 24 hours to examine HSP70 secretion by ELISA. *C*, Macrophages (2×10^5^/well) from LPS-challenged mice were re-stimulated with LPS and IFN-γ at 37°C for 6 hours with or without HSP70 inhibitors: KNK437 (20, 10 µM) or Pifithrin-µ (5, 1 µM) to detect TNF-α production by ELISA. Cells from each treatment condition were pooled from 2–4 mice and measured in triplicate. Data are mean ± SD. Data are representative of two independent experiments. * In comparison of cells with and without HSP70 inhibitors from WBH-mice. ** In comparison of cells with and without HSP70 inhibitors from RT-mice. *, ** *p*<0.05; paired Student *t* test.

We have shown that HT can enhance pro-inflammatory cytokine production by peritoneal macrophages after secondary LPS stimulation. One possibility may involve the induction of heat shock proteins by HT. We hypothesized that HSP70 may be affected since extracellular HSPs, especially HSP70, have immunostimulatory functions that stimulate cytokine production by antigen presenting cells. This effect is mediated by the CD14/TLR4 signaling pathways and the subsequent NF-κB and MAPK activation [29], [30], [31], [32]. To investigate this hypothesis, we evaluated whether HT increased macrophage HSP70 mRNA expression using quantitative real-time PCR (Fig. 5*A*). We found an increase in HSP70 mRNA level in the macrophages from heat-treated mice after *in vitro* LPS/IFN-γ re-stimulation. We then performed ELISA to detect HSP70 secretion in the culture supernatant. We observed that peritoneal macrophages from LPS-challenged, heat-treated mice secreted higher level of HSP70 without *in vitro* re-stimulation as compared to control cells (Fig. 5*B*). In addition, HT and LPS/IFN-γ re-stimulation further increased HSP70 secretion by these macrophages in a time-dependent manner (Fig. 5*B*). We also analyzed macrophage HSP90 mRNA expression and found that HT had no effect on the induction of HSP90 mRNA compared to the cells isolated from control mice (data not shown).

To investigate the direct role of HSP70 on TNF-α production, macrophages from LPS-challenged mice were treated with different concentrations of HSP70 inhibitors (KNK437 or Pifithrin-µ) together with LPS/IFN-γ stimulation. KNK437 inhibits HSP gene transcription by blocking the binding of heat shock factor 1 (HSF1) to the HSP promoter [33]. Pifithrin-µ interacts selectively with the stress-inducible HSP70 protein and inhibits its function [34]. Our results showed that thermally-enhanced TNF-α production was partially-abrogated by treatment with HSP70 inhibitors KNK437 or Pifithrin-µ (Fig. 5*C*) suggesting that heat-induced HSP70 could play a contributing role in mediating thermally-enhanced TNF-α production in macrophages.

### Heat treatment attenuates endotoxin tolerance in peritoneal macrophages

**Figure 6:**
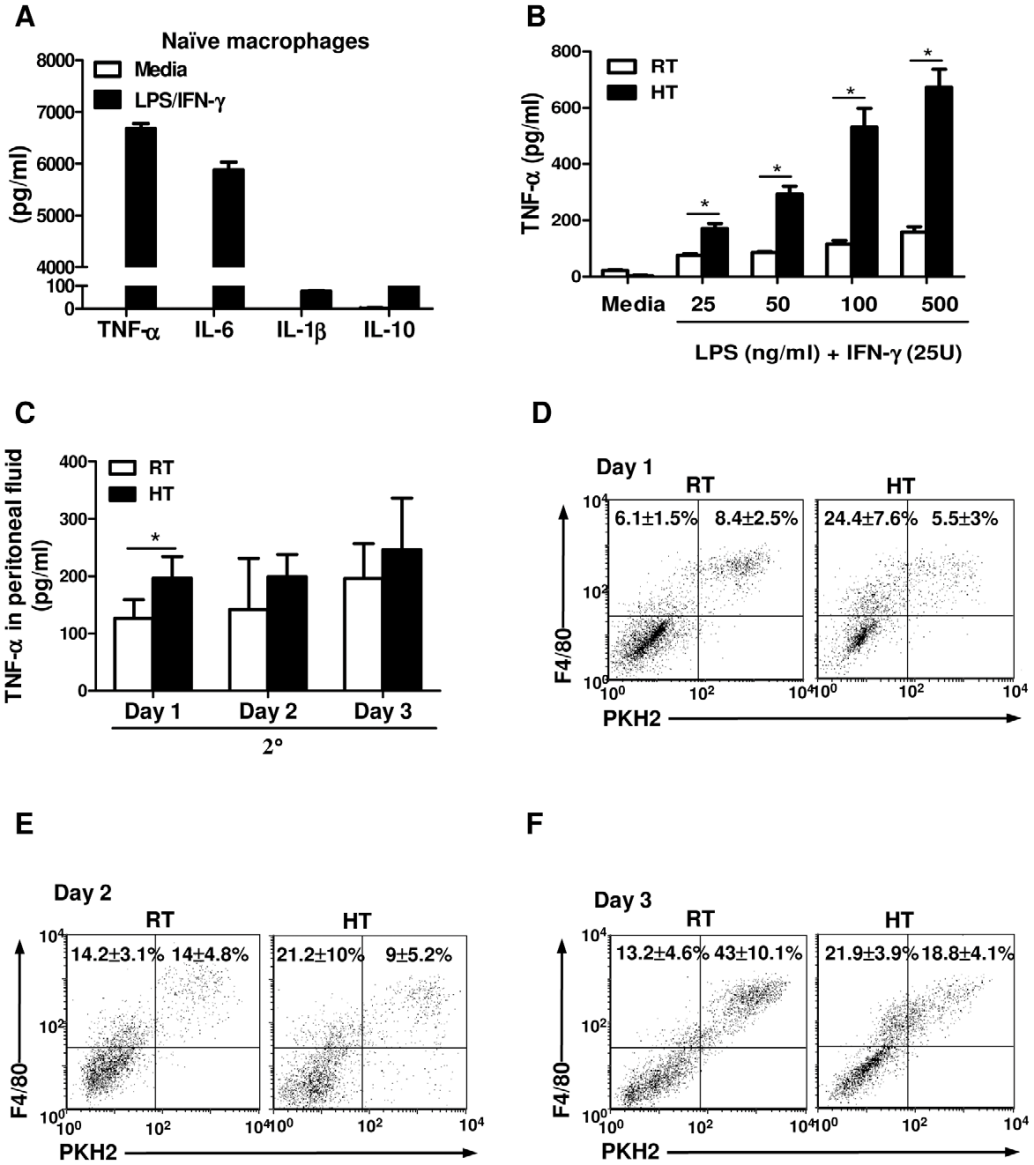
Heat treatment results in a transitory reduction in endotoxin tolerance both *in vitro* and *in vivo*. *A*, Peritoneal macrophages were isolated from naïve mice and stimulated with LPS (100 ng/mL) and IFN-γ (25 U) *in vitro* at 37°C for 6 hours to determine TNF-α, IL-6 and IL-10 or 24 hours for IL-1β production. *B*, Macrophages (2×10^5^/well) were isolated 2 hours post injection from LPS-challenged mice and then re-stimulated with 25 U of IFN-γ and the indicated dose of LPS at 37°C. TNF-α production was determined by ELISA. Cells from each treatment condition were pooled from 2-4 mice and measured in triplicate. Data are mean ± SD. *C*, BALB/c mice were injected intraperitoneally with 10 µg of LPS and then received HT immediately after or were kept at RT for 2 hours on day 0. These mice were then rechallenged with 10 µg of LPS on day 1, 2 or 3. Peritoneal fluids were collected two hours after LPS rechallenge and TNF-α concentration was determined by ELISA. *D to F*, Peritoneal macrophages from mice described in (*C*) were labeled *in vivo* with the fluorescent dye PKH2 two days before the first LPS challenge. These mice were then rechallenged with 10 µg of LPS on day 1 (*D*), 2 (*E*) or 3 (*F*). Peritoneal cells were then collected, stained with F4/80 mAb and analyzed by flow cytometry to determine the percentage of PKH2^+^F4/80^+^ resident and PKH2^-^F4/80^+^ inflammatory macrophages. Each symbol represents an individual mouse (n = 4 mice per treatment groups). Data are mean ± SD. Data are representative of two independent experiments. *p<0.05; paired Student *t* test.

Although heat treatment of LPS-challenged mice increased macrophage pro-inflammatory cytokine production after *in vitro* re-stimulation, the overall amount of cytokine was much less after secondary LPS stimulation (Fig. 2*A*–*C*) as compared to the cells that are only exposed to LPS once (Fig. 6*A*). This phenomenon has been referred to endotoxin tolerance. To characterize whether HT modulates macrophage function and affects endotoxin tolerance, peritoneal macrophages were isolated from LPS-challenged, RT or heat-treated mice and re-stimulated *in vitro* with different concentrations of LPS. Macrophages isolated from RT-mice produced very low amounts of TNF-α in response to all concentrations of LPS used for re-stimulation, which was consistent with previous studies describing LPS-induced *ex vivo* endotoxin tolerance [35], [36]. However, prior HT significantly increased TNF-α production by macrophages being re-stimulated with all doses of LPS, even as low as at 25 ng/mL, and this enhancement was dose-dependent (Fig. 6*B*). Taken together, these data suggest that short term HT of LPS-challenged mice results in downstream changes (seen even after 24 hour recovery period) that endow macrophages with a long lasting ability to increase their production of pro-inflammatory cytokines (although much less than that produced by naïve macrophages) upon LPS re-exposure.

### Heat treatment results in a transitory reduction in endotoxin tolerance *in vivo* and increases the recruitment of inflammatory macrophages

We next wanted to determine whether HT affected the induction of *in vivo* endotoxin tolerance. To test this, BALB/c mice were injected with LPS, received HT or were kept at RT for two hours on day 0 and rechallenged with LPS as a second stimulus on day 1, 2 or 3. TNF-α levels were measured from peritoneal fluids two hours after the second LPS treatment by ELISA (Fig. 6*C*). Consistent with our previous data, HT also reduced endotoxin tolerance *in vivo* by increasing LPS-induced TNF-α secretion into the peritoneal fluid when the mice were rechallenged with LPS on day 1 as compared to the RT mice (Fig. 6*C*). However, this thermal effect was not seen when the mice were rechallenged with LPS on day 2 or 3, suggesting that the thermally-mediated reduction of endotoxin tolerance is transitory.

In addition, we evaluated whether HT affected subsequent inflammatory macrophage recruitment after secondary LPS stimulation. We labeled resident peritoneal macrophages with the fluorescent dye PKH2 two days before the initial LPS challenge. We observed a significant reduction in the percentage of resident macrophages (PKH2^+^ F4/80^+^) in both heated and RT mice (5.5% and 8.4%, respectively) rechallenged with LPS on day 1 (Fig. 6*D*) as compared to mice only exposed to initial LPS stimulation (48%) (Fig. 1*D*). HT significantly increased the percentage of newly recruited inflammatory macrophages (PKH2^−^ F4/80^+^ cells) as compared to control RT mice (24.4% vs. 6.1%) (Fig. 6*D*). However, there was only a modest increase in the cell number of inflammatory macrophages at day 1 (Fig. S5). When the mice were rechallenged with LPS on day 2, there were similar levels of percentage and cell number of resident macrophages in both heat-treated and RT mice. There was a modest increase in the percentage and cell number of newly recruited inflammatory macrophages in the heat-treated group (Fig. 6*E and *S5). In contrast, when we rechallenged these mice with LPS on day 3, HT reduced both the percentage and cell number of resident macrophages without affecting inflammatory macrophage recruitment (Fig. 6*F and *S5). Overall, these data suggest that heat treatment results in a transitory reduction in endotoxin tolerance *in vivo* and increases inflammatory macrophage recruitment.

## Discussion

In this study, we used a mouse model of LPS-induced inflammation to dissect the role of elevated body temperature in regulation of macrophage cytokine production. We found that HT significantly enhanced LPS-induced TNF-α production *in situ* and macrophages were the predominant sources of pro-inflammatory cytokine production in the peritoneal cavity. We also investigated molecular changes induced by HT and found that HT increases the number of TNF-α producing macrophages and enhances TNF-α transcription through increasing LPS-induced NF-κB activation and HSP70 secretion. Importantly, following LPS re-exposure in conjunction with heating, endotoxin tolerance is significantly reduced *in vivo* and *in vitro.* Taken together, our results suggest that induction of a febrile response during inflammation may help to sustain tissue macrophage cytokine production and reduce endotoxin tolerance. This thermally-enhanced pro-inflammatory cytokine production together with other immune cells affected in the thermal microenvironment may produce synergistic effects and be beneficial for the host to eliminate pathogens and accelerate the resolution process of inflammation. However, it is important to recognize that the model we have used here, while extremely useful, is an aseptic model and should be distinguished from a septic, or infectious disease models (e.g., Steiner et al. [37] and Guptill et al. [38]). Whether elevated temperature has similar effects on macrophage function or endotoxin tolerance during infectious disease is not yet clear and should be investigated.

By using chimeric mice, Steiner et al. have demonstrated that the early phase of LPS-induced fever is triggered exclusively by TLR4-expressing macrophages of the major LPS-processing organs, the liver and lung, whereas later phases of fever depend on both hematopoietic and non-hematopoietic TLR4-positive cells [39], [40]. Fever is initiated by circulating PGE2 synthesis by these macrophages via up-regulation of COX-2 [41]. Because febrile response requires high energy cost, Romanovsky et al. have proposed that fever is an adaptive thermoregulatory response to systemic inflammation and is beneficial only when there is no immediate threat of energy deficit [42], [43]. A natural fever in mice and humans, and many other species, is generated not only by metabolic changes, but also a strong “heat seeking” behavior, causing the individual to move to, or create warmer environments that help to sustain an increased body temperature [11], [44], [45], [46]. Studies by Rudaya et al. have shown that LPS-induced systemic inflammation and febrile response in mice largely depends on ambient temperature and LPS dose [47], while studies by others show that there is only minor LPS-induced natural fever when mice are housed under relatively cool standard room temperature conditions required in animal research colonies [46], [48], [49], [50]. Our results confirm that there is only a slight elevation of body temperature of 1°C or less after LPS injection in mice maintained under standard housing temperature necessitating the need for additional ambient warmth to achieve higher temperatures. Since achieving fever-range temperatures during a natural fever in mice is due to both metabolic changes as well as through a “behavioral fever” or “heat seeking” migration to warmer ambient temperatures, placing mice in a warmer ambient temperature following endotoxin challenge (e.g., LPS) to study the role of increased body temperature can be considered a physiologically relevant procedure [51], [52]. By using this procedure, we found that HT significantly enhanced LPS-induced TNF-α production *in situ*. Since we found *less* intracellular TNF-α in macrophages isolated from LPS-challenged, heat-treated mice as compared to cells from RT mice, we interpreted this result to be due to the fact that HT has increased LPS-induced TNF-α secretion from macrophages. Indeed, since these macrophages secreted cytokines shortly after LPS stimulation, the remaining intracellular staining of TNF-α seen after isolation would represent only a small fraction due to new synthesis.

HT also helped inflammatory macrophage recruitment following LPS stimulation, which may help pathogen clearance to accelerate resolution of inflammation. Since inflammatory cytokines are very important in limiting disease progression and survival in the infected host is enhanced by early production of pro-inflammatory cytokines, such as TNF-α and IL-1β [53], our results suggested that the thermal exposure might provide a significant survival advantage for the host following infection.

To further determine how thermal microenvironment could affect macrophage function, we isolated peritoneal macrophages from the LPS-challenged mice and re-stimulated these cells *in vitro* with LPS/IFN-γ at 37°C. We observed that peritoneal macrophages from LPS-challenged mice *which had received prior HT* produced significantly higher amounts of TNF-α, IL-6 and IL-1β (long after the heating has been completed) as compared to cells from the mice maintained at RT. Normally, when peritoneal macrophages are pre-activated *in vivo* by LPS, they are not as responsive to a subsequent *in vitro* re-stimulation as cells are after an initial LPS exposure. This change is due to an induction of “endotoxin tolerance.” Endotoxin tolerance is a phenomenon in which the host that is pre-exposed to LPS exhibits a suppressed production of cytokines when challenged again with the same antigen *in vivo* and *in vitro* [54], [55], [56], [57]. Monocytes/macrophages have been shown to be the principal cells involved in the induction of endotoxin tolerance [58], [59]. Endotoxin tolerance serves to dampen excessive inflammation [60], [61]. While *ex vivo* endotoxin tolerance quickly resolves (24 hours), *in vivo* endotoxin tolerance can persist for weeks [55]. Although endotoxin tolerance is a critical host defensive system, the clinical relevance of this reaction is difficult to demonstrate in patients [62], [63], [64] and there are no conclusive results to support the relationship between endotoxin tolerance and host survival [65], [66], [67], [68], [69]. Moreover, there are also concerns that this hyporesponsive state may render the host vulnerable for secondary infection and increase mortality [59], [70]. Indeed, immune cells may experience continuous antigen exposure before the pathogens are eliminated during inflammation. How the immune system adapts to endotoxin tolerance and still be able to respond to antigen re-exposure is poorly understood. Importantly, our study demonstrated that short HT could reduce LPS-induced endotoxin tolerance and maintain the responsiveness of macrophages for subsequent LPS rechallenge. This finding suggests that local thermal microenvironment could play an important role in the extent of endotoxin tolerance. Interestingly, a previous study shows that vagal innervation of the liver (a route for the transduction of systemic inflammatory/pyrogenic signals to the brain, helping to raise body temperature) is required for the development of endotoxin tolerance [71]. These data suggest that regulation of endotoxin tolerance may be quite complex and dependent upon various local microenvironmental factors.

As shown by Steiner et al, inflammatory signaling (e.g. NF-κB activation measured by a decrease in the content of IκB-α) was activated in the lung and liver at the onset of LPS-induced fever (40 minutes after LPS injection) [41]. Therefore, NF-κB signaling might be the potential molecular target of the thermal microenvironment on macrophage activation. Our results support the involvement of NF-κB in heat-treated macrophages through an increase of IKK and IκB phosphorylation, NF-κB nuclear translocation and NF-κB binding to the TNF-α promoter region. Murine TNF-α promoter contains 4 κB binding sites located at 210, 510, 655 and 850 nucleotide upstream of the transcription start site. However, the specific contribution of each binding region is not fully understood [28]. Therefore, understanding why mild heating appeared to only enhance NF-κB binding to the regions −364/−182 and −685/−543 or whether there is any site-specific regulation will need further investigation.

We have determined here that HT can reprogram tissue resident macrophages for a more longstanding enhanced pro-inflammatory cytokine production, and we wondered about potential mechanisms. One possibility may involve the induction of heat shock proteins by heat treatment. HSPs can be released from various cells through either a passive (from necrotic cells after injury) or an active (translocation to the plasma membrane and then secretion) pathway [30]. Extracellular HSPs, especially HSP70 are reported to stimulate the release of TNF-α, IL-6, IL-1β, IL-12, nitric oxide as well as chemokines by monocytes, macrophages and dendritic cells [29], [30]. This effect is mediated by the CD14/TLR4 signaling pathways [29], [31], [32]. Therefore, HSPs may serve as endogenous danger signals to alert the host defense system through their cytokine-like functions [30], [72], [73]. We found a significant increase in HSP70 mRNA induction after *in vitro* LPS/IFN-γ re-stimulation in the cells isolated from LPS-challenged, heat-treated mice. We also detected that more HSP70 was secreted from these cells in a time-dependent manner. This extracellular HSP70 may play a role in enhancing LPS-induced TNF-α production because this thermal effect was abrogated in the presence of HSP70 inhibitors (KNK437 and Pifithrin-µ). However, HSP70 inhibitors only show partial abrogation of this thermally-enhanced TNF-α production. This indicates that there must be other thermally-induced mediators that also play a role in regulating macrophage TNF-α production. Overall, these data suggest that prior HT enhances HSP70 secretion from macrophages after secondary LPS stimulation which may, in a paracrine or autocrine fashion, help to enhance the signals from LPS and promote TNF-α production through binding to TLR4.

Collectively, these results provide novel insights into the role of febrile temperature in regulation of resident macrophage function and reduction of endotoxin tolerance during inflammation. We also identify potential cellular targets of thermal signals which may help to explain how heating increases LPS-induced pro-inflammatory cytokine production *in vivo* and *in vitro*. Finally, although we demonstrate here that HT can positively regulate naïve macrophage cytokine production, several studies have revealed a strong anti-inflammatory effect of HT using the long-term macrophage cell line RAW264.7 or human monocyte-derived macrophages [74], [75], [76] heated *in vitro*. Since RAW cells are considered to be in a state of chronic activation, it is possible that the effects of temperature are dependent upon the activation stage of macrophages. These data demonstrate that more study is needed to identify precise thermally sensitive mechanisms by which physiological temperature shifts regulate cellular functions in order to fully understand microenvironmental contributions to macrophage function.

## Materials and Methods

### Ethics statement

Eight to nine-week-old female BALB/c mice (NCI) were housed under pathogen-free conditions at Roswell Park Cancer Institute (RPCI) and were used in all experiments with age-matched control. All animal procedures were performed in strict accordance with the recommendations in the Guide for the Assessment and Accreditation of Laboratory Animal Care International (AALAC). The protocol was approved by the Institutional Animal Care and Use Committee at RPCI (Protocol number: 797M).

### LPS administration and heat treatment (HT)

BALB/c mice were injected intraperitoneally with 10 µg LPS (Escherichia coli serotype O111:B4, Sigma-Aldrich) in 1 mL sterile saline. Mice were immediately placed in microisolator cages preheated to 36.5°C in a gravity convection oven (Memmert model BE500, Wisconsin Oven). Mice core body temperatures were raised within 20 min and then maintained at 39.0°C (±0.4°C) for two hours by adjusting the incubator temperature. Mouse core body temperature in each cage was monitored with the Electronic Laboratory Animal Monitor System using mice that had microchip transponder (Bio Medic Data Systems, Delaware, USA) subcutaneously implanted into the dorsal thoracic area. Control mice were kept at room temperature (RT, ∼22–24°C) and subjected to the same handling.

### Isolation and stimulation of peritoneal macrophages

Peritoneal cells were harvested from heat-treated or control mice 2 hours post LPS administration by peritoneal lavage with 6 mL cold PBS and collected by centrifugation. Macrophages were enriched by adherence to plastic in complete media (RPMI containing 10% fetal calf serum and 100 U/mL penicillin/streptomycin, Cellgro) for 1 hour and recovered overnight to prevent the non-specific stress response induced by the isolation. Macrophages were then re-stimulated with 100 ng/mL LPS (Escherichia coli serotype O111:B4, Sigma-Aldrich) and 25 U IFN-γ (R&D systems) at 37°C for indicated time points. Supernatant was collected to determine the concentrations of TNF-α, IL-1β, IL-10 (Biolegend), IL-6 (BD Biosciences) and HSP70 (R&D systems) by ELISA. To detect TNF-α production at the single cell level, mouse TNF-α/TNFSF1A ELISpot was performed according to the manufacturer's instructions (R&D Systems). In some experiments, macrophages were treated with HSP70 inhibitors (KNK437, EMD chemicals; Pifithrin-µ, Sigma-Aldrich) together with 100 ng/mL LPS and 25 U IFN-γ.

### *In vivo* labeling of resident peritoneal macrophages

Peritoneal macrophages were selectively labeled *in situ* by injecting 0.5 mL of the diluted fluorescent dye PKH2 (Sigma-Aldrich) intraperitoneally into BALB/c mice two days before the LPS challenge. Mice were then injected intraperitoneally with 10 µg LPS and immediately received HT or were kept at RT for 2 hours. Labeled peritoneal cells were harvested from heat-treated or control mice and incubated with mAbs specific for mouse F4/80 (BM8-PE; BioLegend) then analyzed by flow cytometry (FacsCalibur, BD Biosciences). Analysis of data was performed with FCS Express (De Novo Software).

### *In vivo* endotoxin tolerance induction and WBH treatment

*In vivo* endotoxin tolerance was induced in BALB/c mice by initially injecting 10 µg LPS (Escherichia coli serotype O111:B4, Sigma-Aldrich) intraperitoneally in 1 mL sterile saline on day 0. These mice were received HT immediately after LPS injection or were maintained at RT for two hours. They were then rechallenged with 10 µg LPS as a second stimulus on day 1, 2 or 3. Peritoneal fluids were collected by peritoneal lavage with 1 mL cold PBS two hours after the second LPS treatment to determine the concentrations of TNF-α by ELISA (Biolegend).

### Intracellular cytokine staining

Peritoneal cells collected from LPS-challenged mice were stained with FITC-conjugated anti-CD11b (BD Biosciences), PE-conjugated anti-TNF-α Ab (BioLegend) by the BD Cytofix/Cytoperm fixation/Permeabilization kit (BD biosciences) and analyzed by flow cytometry (FacsCalibur, BD Biosciences). Analysis of data was performed with FCS Express (De Novo Software).

### RNA extraction and **quantitative** real-time PCR

Peritoneal macrophages collected from LPS-challenged, heat-treated or RT mice were re-stimulated with LPS and IFN-γ at 37°C for 4 hours. The cells were lysed and total RNA was extracted using the RNeasy kit (QIAGEN). One microgram of RNA was reverse transcribed using oligo (dT) and Superscript III first strand synthesis system (Invitrogen). Resulting cDNA was used as template to analyze the expression of target genes by quantitative real-time PCR using specific primers and SYBR green master mix (Roche). The results of real time-PCR were then analyzed using an Applied Biosystems real-time PCR system (ABI 7900 HT). Data were generated with the comparative threshold cycle (ΔΔCT) method and normalized to the housekeeping gene GAPDH. The following primers were used: TNF-α forward, caccacgctcttctgtctactgaact; reverse, gggctacaggcttgtcactcgaattt; HSP70 forward, agcgaggctgacaagaagaaggt; reverse, accctggtacagcccactgatgat.

### Measurement of NF-κB nuclear translocation

Peritoneal macrophages from LPS-challenged, heat-treated or RT mice were stimulated with LPS and IFN-γ at 37°C for 15, 30 and 60 minutes and then incubated with mAbs for mouse CD11b (M1/70-PE; BD Biosciences) and fixed with 4% formaldehyde (Polysciences, Inc). The cells were then incubated with mAb for NF-κB p65 (F-6-FITC; Santa Cruz biotechnology) in permeabilization wash buffer (3% fetal calf serum, 0.1% 100X Triton-X) and analyzed by ImageStream flow cytometry (Amnis). mAb for DRAQ5 (Cell Signaling) was used for nuclear staining. We quantified the degree of NF-κB nuclear translocation by calculating the similarity of NF-κB and nuclear dye DRAQ5 images in CD11b^+^ macrophages. Cells with low similarity scores exhibit no correlation between the images (corresponding with a predominant cytoplasmic distribution of NF-κB), whereas cells with high similarity scores exhibit a positive correlation between the images (corresponding with a predominant nuclear distribution of NF-κB). The relative shift in this distribution between two populations (e.g., untreated versus treated cells) was calculated using the Fisher's Discriminant ratio (*R*_d_ value) [26]. *R*_d_ value was calculated based on the formula: *R*_d_ value = (similarity score of treated-untreated)/(standard deviation of the similarity score from treated+ untreated).

### Preparation of cell extracts and Western blotting analysis

Cell extracts were prepared in lysis buffer (containing 0.5 M Tris, 2.5 M NaCl, 500 mM NaF and 10% nonionic P_40_ with protease inhibitors: 200 mM Na_3_VO_4_, 0.5 M β-glycerophosphate, 0.25 M NaPPi, 0.1 M PMSF, 1 mg/mL leupeptine, 0.1 M benzamidine and 1 mg/mL aprotinin). For Western blotting analysis, 40 µg total protein lysates were resolved by SDS-PAGE, transferred to a polyvinylidene difluoride membrane (Millipore) and blocked with either 5% nonfat milk in PBS-T or 3% BSA in TBS-T. The membrane was then probed with primary antibodies specific for phosphorylated (phospho) IKK and phospho-IκB (Cell Signaling) followed by horseradish peroxidase-conjugated secondary antibody and developed with ECL-detecting reagents (Thermo Scientific). The band intensity was quantified using Scion Image (Scion Corporation).

### Chromatin immunoprecipitation (ChIP) assay

Peritoneal macrophages collected from LPS-challenged, heat-treated or RT mice were re-stimulated with LPS and IFN-γ at 37°C for 1 hour, cross-linked with 1% formaldehyde and collected by centrifugation. The cell pellets were resuspended in SDS lysis buffer with protease inhibitors (Thermo Scientific) and sonicated for five 10 seconds burst using Sonic Dismembrator model 300 (Fisher). Sonicated cell lysates were precipitated with 5 µg anti-NFκB p65 (Abcam) or control antibody (normal rabbit IgG, Cell Signaling) at 4°C overnight. The immune complexes were collected with ChIP-grade protein G magnetic beads (Cell Signaling). Cross-linked protein-DNA was then eluted and reverted by incubating at 65°C overnight. DNA was extracted, used as template for quantitative real-time PCR and then analyzed using an Applied Biosystems real-time PCR system (ABI 7900 HT). Data were generated with the comparative threshold cycle (ΔΔCT) method and normalized to the input and control IgG. The following primers were used for TNF promoter: −912/−763: forward, gagaagtgactccactggagggt; reverse, actgcggtacatcaactcagacat; −685/−543: forward, aaggcttgtgaggtccgtga; reverse, aagtggctgaaggcagagca; −586/−468: forward, acttcccaactctcaagctgctct; reverse, gtgcttctgaaagctgggtgcata; −364/−182: forward, tctggaggacagagaagaaatg; reverse, ggtttggaaagttggggacac.

### Statistic analysis

Data were analyzed with the Student's *t* tests to determine statistical significance. *P* values of less than 0.05 were considered to represent statistically significant differences.

## Supporting Information

Figure S1 **Total cell number of resident and inflammatory macrophages after LPS stimulation.** *A* to *B*, Cell numbers of PKH2^+^F4/80^+^ resident (*A*) and PKH2^−^F4/80^+^ inflammatory macrophages (*B*) 1 and 2 days after LPS injection. Each symbol represents an individual mouse.
(TIF)

Figure S2 **Effect of heat treatment on TNF-α production by naïve macrophages.** Peritoneal macrophages were isolated from naïve mice with or without 2 hour heat treatment and stimulated with LPS/IFN-γ *in vitro* at 37°C for 6 hours to determine TNF-α by ELISA. Cells from each treatment condition were pooled from 2 mice and measured in triplicate. Data are mean ± SD.
(TIF)

Figure S3 **Effect of heat treatment on TLR4 expression on the surface of macrophages.** Peritoneal macrophages were isolated from naïve and LPS-challenged, RT or heated mice. These cells were stained with antibodies against CD11b and TLR4 and analyzed by flow cytometry. Each symbol represents the MFI of TLR4 from an individual mouse.
(TIF)

Figure S4 **Binding of NF-κB to TNF-α promoter in naïve macrophages after LPS/IFN-γ stimulation.** ChIP assay and quantitative real-time PCR were used to analyze NF-κB p65 binding to TNF-α promoter regions −364/−182, −586/−468, −685/−543 and −912/−763 in naïve macrophages after LPS/IFN-γ stimulation. The graph shows the fold change that is normalized to the input, control IgG and unstimulated control.
(TIF)

Figure S5 **Total cell number of resident and inflammatory macrophages after LPS re-exposure.** *A* to *C*, Cell numbers of PKH2^+^F4/80^+^ resident and PKH2^−^F4/80^+^ inflammatory macrophages 1, 2 and 3 days after *in vivo* LPS rechallenge. Each symbol represents an individual mouse. Data are mean ± SD. *p<0.05; paired Student *t* test.
(TIF)
