# Supplementary figures and images for: Elevation in Body Temperature to Fever Range Enhances and Prolongs Subsequent Responsiveness of Macrophages to Endotoxin Challenge
Source: PLoS One. 2012 Jan 10;7(1):e30077. doi: 10.1371/journal.pone.0030077 (PMC3254634; doi:10.1371/journal.pone.0030077)

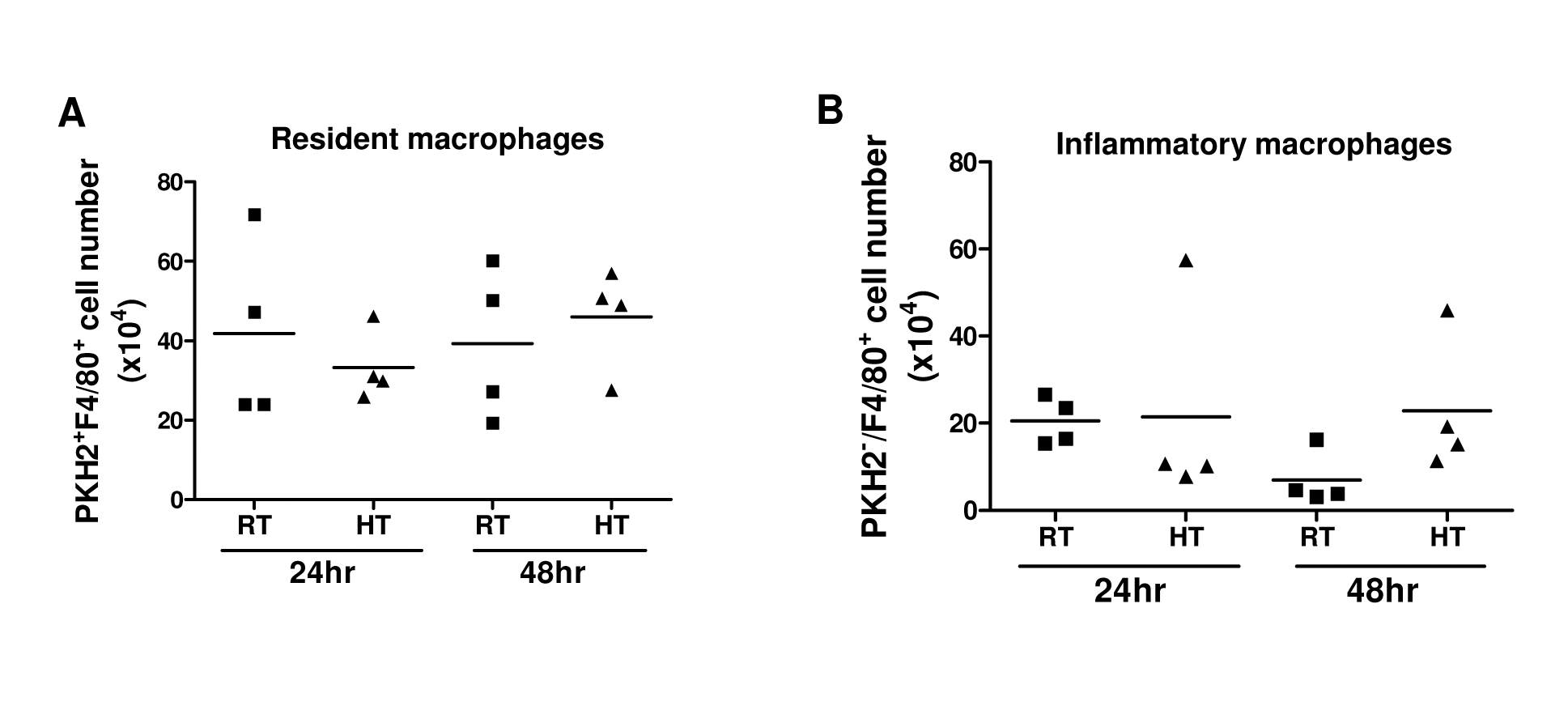

Supplement: Figure S1 — Total cell number of resident and inflammatory macrophages after LPS stimulation. A to B, Cell numbers of PKH2+F4/80+ resident (A) and PKH2−F4/80+ inflammatory macrophages (B) 1 and 2 days after LPS injection. Each symbol represents an individual mouse. (TIF) [file pone.0030077.s001.tif]

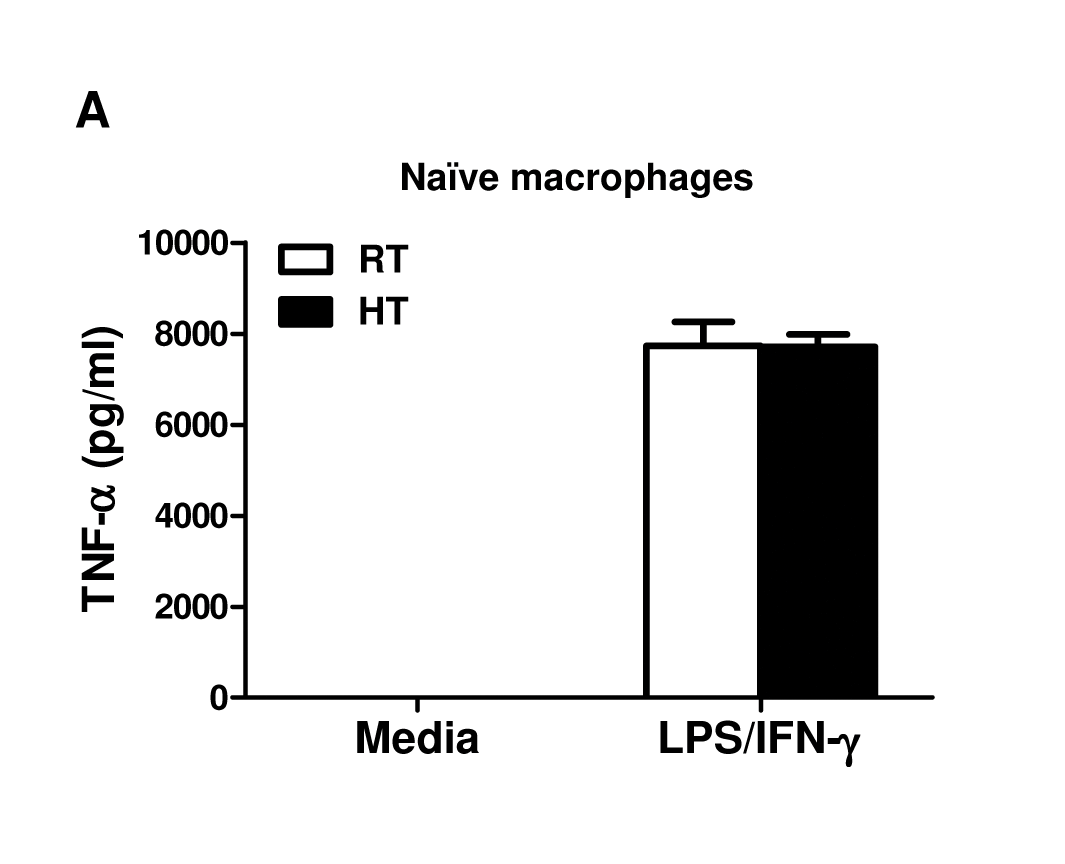

Supplement: Figure S2 — Effect of heat treatment on TNF-α production by naïve macrophages. Peritoneal macrophages were isolated from naïve mice with or without 2 hour heat treatment and stimulated with LPS/IFN-γ in vitro at 37°C for 6 hours to determine TNF-α by ELISA. Cells from each treatment condition were pooled from 2 mice and measured in triplicate. Data are mean ± SD. (TIF) [file pone.0030077.s002.tif]

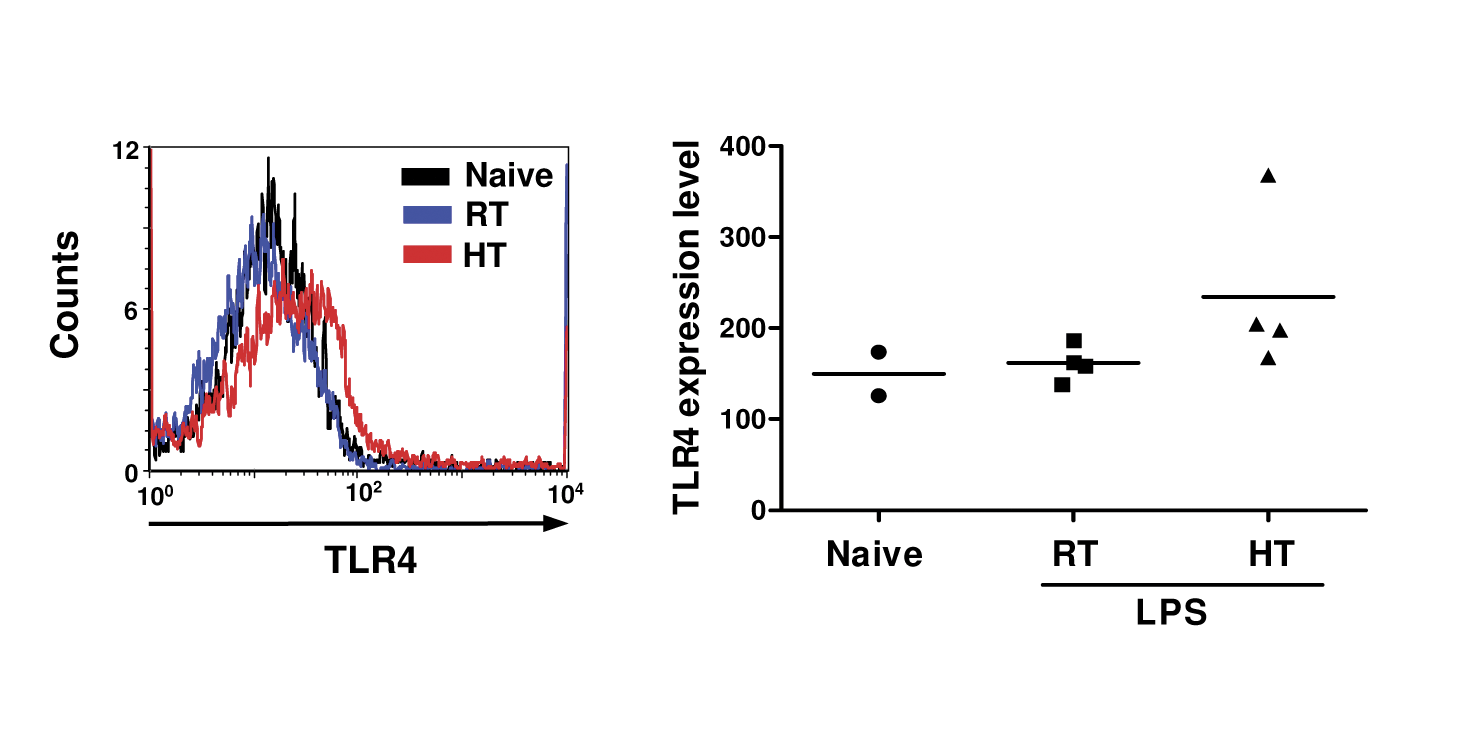

Supplement: Figure S3 — Effect of heat treatment on TLR4 expression on the surface of macrophages. Peritoneal macrophages were isolated from naïve and LPS-challenged, RT or heated mice. These cells were stained with antibodies against CD11b and TLR4 and analyzed by flow cytometry. Each symbol represents the MFI of TLR4 from an individual mouse. (TIF) [file pone.0030077.s003.tif]

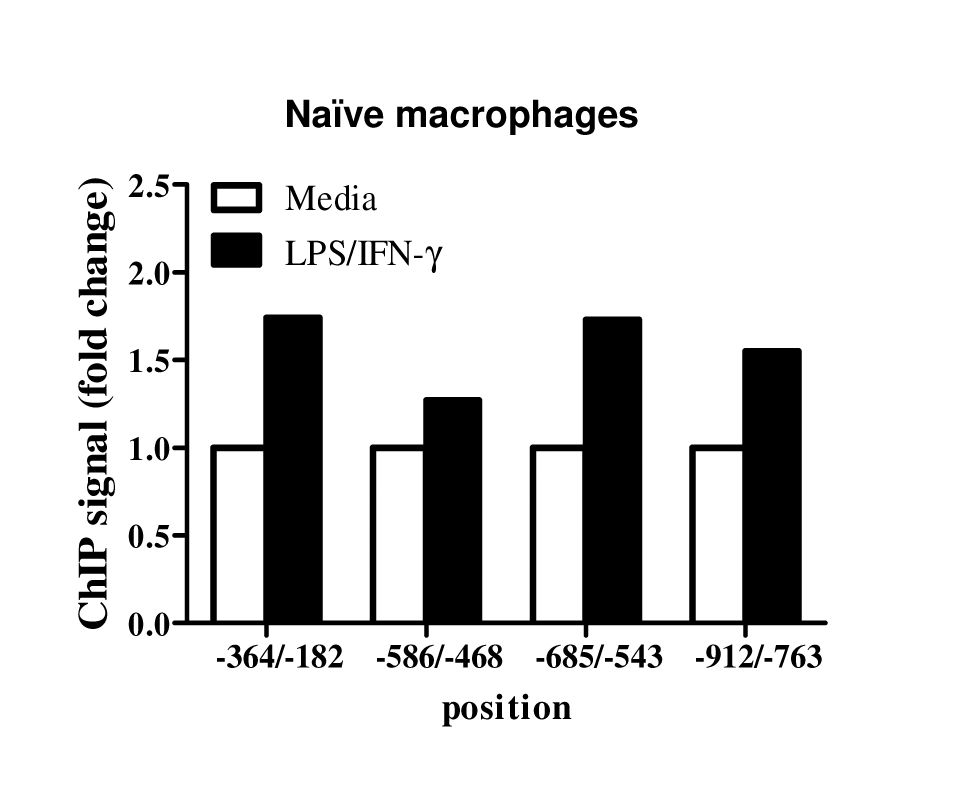

Supplement: Figure S4 — Binding of NF-κB to TNF-α promoter in naïve macrophages after LPS/IFN-γ stimulation. ChIP assay and quantitative real-time PCR were used to analyze NF-κB p65 binding to TNF-α promoter regions −364/−182, −586/−468, −685/−543 and −912/−763 in naïve macrophages after LPS/IFN-γ stimulation. The graph shows the fold change that is normalized to the input, control IgG and unstimulated control. (TIF) [file pone.0030077.s004.tif]

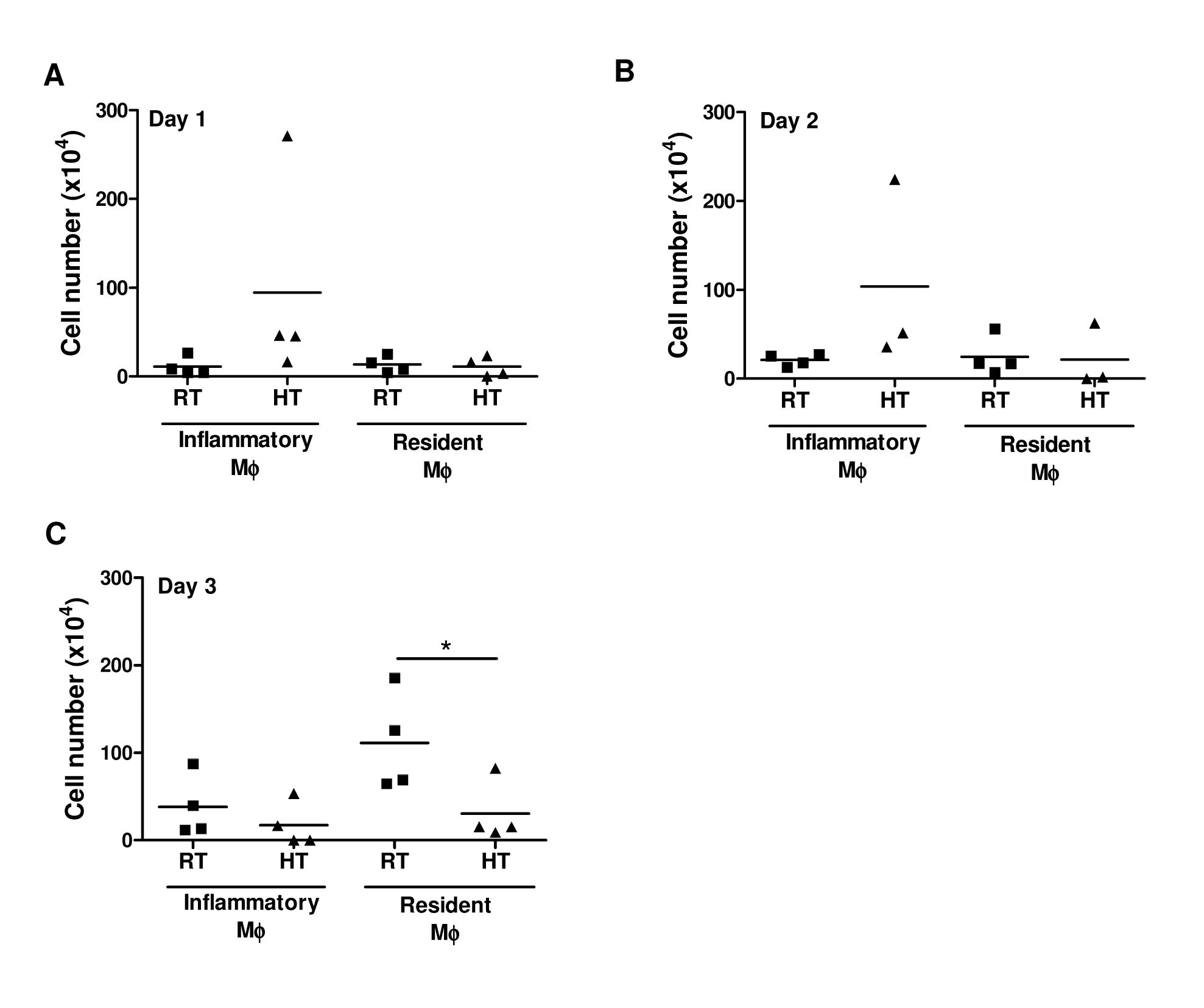

Supplement: Figure S5 — Total cell number of resident and inflammatory macrophages after LPS re-exposure. A to C, Cell numbers of PKH2+F4/80+ resident and PKH2−F4/80+ inflammatory macrophages 1, 2 and 3 days after in vivo LPS rechallenge. Each symbol represents an individual mouse. Data are mean ± SD. *p<0.05; paired Student t test. (TIF) [file pone.0030077.s005.tif]
